# Suicide Among Adults Aged 35–64 Years — United States, 1999–2010

**Published:** 2013-05-03

**Authors:** Erin M. Sullivan, Joseph L. Annest, Feijun Luo, Thomas R. Simon, Linda L. Dahlberg

**Affiliations:** Div of Analysis, Research, and Practice Integration; Div of Violence Prevention, National Center for Injury Prevention and Control, CDC

Suicide is an increasing public health concern. In 2009, the number of deaths from suicide surpassed the number of deaths from motor vehicle crashes in the United States (1). Traditionally, suicide prevention efforts have been focused mostly on youths and older adults, but recent evidence suggests that there have been substantial increases in suicide rates among middle-aged adults in the United States (2). To investigate trends in suicide rates among adults aged 35–64 years over the last decade, CDC analyzed National Vital Statistics System (NVSS) mortality data from 1999–2010. Trends in suicide rates were examined by sex, age group, race/ethnicity, state and region of residence, and mechanism of suicide. The results of this analysis indicated that the annual, age-adjusted suicide rate among persons aged 35–64 years increased 28.4%, from 13.7 per 100,000 population in 1999 to 17.6 in 2010. Among racial/ethnic populations, the greatest increases were observed among American Indian/Alaska Natives (AI/ANs) (65.2%, from 11.2 to 18.5) and whites (40.4%, from 15.9 to 22.3). By mechanism, the greatest increase was observed for use of suffocation (81.3%, from 2.3 to 4.1), followed by poisoning (24.4%, from 3.0 to 3.8) and firearms (14.4%, from 7.2 to 8.3). The findings underscore the need for suicide preventive measures directed toward middle-aged populations.

CDC used the Web-based Injury Statistics Query and Reporting System (3) to compile NVSS data on suicides reported during 1999–2010 among U.S. residents aged ≥10 years. Age group–specific annual suicide rates, as well as age-adjusted annual suicide rates calculated using the U.S. standard 2000 population, were based on bridged race population estimates from the U.S. Census Bureau. Trends in age-adjusted suicide rates from 1999, when signs of an increase began (4), through 2010, the latest data available, were analyzed for adults aged 35–64 years by sex and mechanism of suicide. The three most common suicide mechanisms were firearms (i.e., penetrating injury or gunshot wound from a weapon using a powder charge to fire a projectile), poisoning (predominantly drug overdose), and suffocation (predominantly hanging). These three mechanisms and an “all other” mechanism category were used for comparisons. Data also were analyzed by age group, race/ethnicity,* and U.S. Census region.

Percentage changes in observed suicide rates from 1999 to 2010 were calculated along with corresponding 95% confidence intervals, assuming a Poisson distribution. Tests of significance of trends in annual age-adjusted suicide rates for adults aged 35–64 years across the 12-year period were conducted using joinpoint regression (5), assuming a log-linear model. This report focuses on adults aged 35–64 years because percentage changes from 1999 to 2010 in the annual age-adjusted suicide rates for persons aged 10–34 years and ≥65 years were comparatively small and not statistically significant (a 7.0% increase from 9.2 in 1999 to 9.9 in 2010 [p = 0.06] and a 5.9% decrease from 15.8 in 1999 to 14.9 in 2010 [p = 0.09], respectively). Finally, data were analyzed by state, and percentage changes in age-adjusted suicide rates from 1999 to 2010 were calculated for all 50 states.

From 1999 to 2010, the age-adjusted suicide rate for adults aged 35–64 years in the United States increased significantly by 28.4%, from 13.7 per 100,000 population to 17.6 (p<0.001) (Table 1). The suicide rate for men aged 35–64 years increased 27.3%, from 21.5 to 27.3, and the rate for women increased 31.5%, from 6.2 to 8.1 (Table 2). Among men, the greatest increases were among those aged 50–54 years and 55–59 years, (49.4%, from 20.6 to 30.7, and 47.8%, from 20.3 to 30.0, respectively). Among women, suicide rates increased with age, and the largest percentage increase in suicide rate was observed among women aged 60–64 years (59.7%, from 4.4 to 7.0).

What is already known on this topic?

Traditionally, suicide prevention efforts have been focused mostly on youths and older adults, but recent evidence suggests that suicide rates among middle-aged adults in the United States have increased substantially. Firearms, suffocation (predominantly hanging), and poisoning (predominantly drug overdose) are the three leading mechanisms of suicide in the United States.

What is added by this report?

The annual, age-adjusted suicide rate among persons aged 35–64 years increased 28.4%, from 13.7 per 100,000 population in 1999 to 17.6 in 2010. Among racial/ethnic populations, the greatest increases were observed among American Indian/Alaska Natives (65.2%, from 11.2 to 18.5) and whites (40.4%, from 15.9 to 22.3). By mechanism, the greatest rate increase was observed for suffocation (81.3%, from 2.3 to 4.1), followed by poisoning (24.4%, from 3.0 to 3.8) and firearms (14.4%, from 7.2 to 8.3). Significant increases were observed across all regions in the United States.

What are the implications for public health practice?

These results highlight the need for suicide prevention strategies that address mental health issues and the stresses and challenges that middle-aged adults are likely to face. Such stresses include economic challenges, dual caregiver responsibilities (children and aging parents), and potential health problems.

By racial/ethnic population, the greatest increases from 1999 to 2010 among men and women overall were observed among AI/ANs (65.2%, from 11.2 to 18.5) and whites (40.4%, from 15.9 to 22.3). Among AI/ANs, the suicide rate for women increased 81.4%, from 5.7 to 10.3; the rate for men increased 59.5%, from 17.0 to 27.2. Among whites, the rate for women increased 41.9%, from 7.4 to 10.5; the rate for men increased 39.6%, from 24.5 to 34.2.

Suicide rates from 1999 to 2010 increased significantly across all four geographic regions and in 39 states.† In 2010, rates for adults aged 35–64 years were highest (19.5 per 100,000 population) in the West U.S. Census Region (Table 1). By suicide mechanism, age-adjusted rates increased for the three primary mechanisms for both men and women (Figure). Firearms and suffocation were the most common mechanisms for men (14.3 and 6.8 in 2010, respectively), whereas poisoning and firearms were the most common mechanisms for women (3.4 and 2.5 in 2010, respectively). By mechanism, the greatest increase was observed for use of suffocation (81.3%, from 2.3 to 4.1), followed by poisoning (24.4%, from 3.0 to 3.8) and firearms (14.4%, from 7.2 to 8.3) (Table 1). By sex, the increase for suffocation was 75.0% for men (from 3.9 to 6.8) and 115.0% for women (from 0.7 to 1.5) (Table 2). From 1999 to 2010, suicides by suffocation increased from 18% to 24% of all suicides for men and from 12% to 18% of all suicides for women.

## Editorial Note

Suicide rates among both men and women aged 35–64 years increased substantially from 1999 and 2010. This finding is consistent with a previous study that showed a notable increase in the overall suicide rate among middle-aged adults relative to a small increase in suicide rates among younger persons and a small decline in older persons during a similar period (2). The increases were geographically widespread and occurred in states with high, as well as average and low suicide rates. By race/ethnicity, the increases were highest and statistically significant only among whites and American Indian/Alaska Natives, widening the racial/ethnic gap in suicide rates (3).

Prevalence of mechanisms of suicide changed from 1999 to 2010. Whereas firearm and poisoning suicide rates increased significantly, suffocation (predominantly hanging) suicide rates increased the most among men and women aged 35–64 years. This increasing trend is particularly troubling because a large proportion of suicide attempts by suffocation result in death, suggesting a need for increased public awareness of suicide risk factors and research of potential suicide prevention strategies to reduce suffocation deaths (2).

Possible contributing factors for the rise in suicide rates among middle-aged adults include the recent economic downturn (historically, suicide rates tend to correlate with business cycles, with higher rates observed during times of economic hardship) (6,7); a cohort effect, based on evidence that the “baby boomer” generation had unusually high suicide rates during their adolescent years (8); and a rise in intentional overdoses associated with the increase in availability of prescription opioids (1,2). Additional research is needed to understand the cause of the increase in age-adjusted suicide rates and why the extent of the increase varies across racial/ethnic populations.

The findings in this report are subject to at least four limitations. First, the findings are subject to variation among state coroners/medical examiners regarding determination of manner of death, especially for poisoning, as recorded on the death certificate (9). Second, suicide rates likely are an underestimate of the actual prevalence because suicides might be undercounted in NVSS (9). Third, NVSS lacks information about factors such as physical and mental health history at the time of suicide and recent stressors that might have contributed to risk for suicide. The National Violent Death Reporting System collects more comprehensive information on suicide circumstances but the system currently is limited to 18 states.§ Finally, suicide rates might be affected by death certificate race/ethnicity misclassification, particularly for AI/ANs.¶

Most suicide research and prevention efforts have focused on youths and older adults. Although the analysis in this report does not explain why suicide rates are increasing so substantially among middle-aged adults, the results underscore the importance of prevention strategies that address the needs of persons aged 35–64 years, which includes the baby boomer cohort. Prevention efforts are particularly important for this cohort because of its size, history of elevated suicide rates, and movement toward older adulthood, the period of life that has traditionally been associated with the highest suicide rates (3,8).

The 2012 Surgeon General’s *National Strategy for Suicide Prevention* describes salient risk factors, prevention opportunities, and existing resources to help those at increased risk for suicide (10). Suicide prevention strategies such as those that enhance social support, community connectedness, and access to mental health and preventive services, as well as efforts to reduce stigma and barriers associated with seeking help, are important for addressing suicide risk across the lifespan. Other strategies are likely to be particularly critical for addressing the needs of middle-aged adults, such as those that help persons overcome risk factors, which include economic challenges, job loss, intimate partner problems or violence, the stress of caregiver responsibilities (often for children and aging parents), substance abuse, and declining health or chronic health problems (7,8,10).

## Figures and Tables

**FIGURE:**
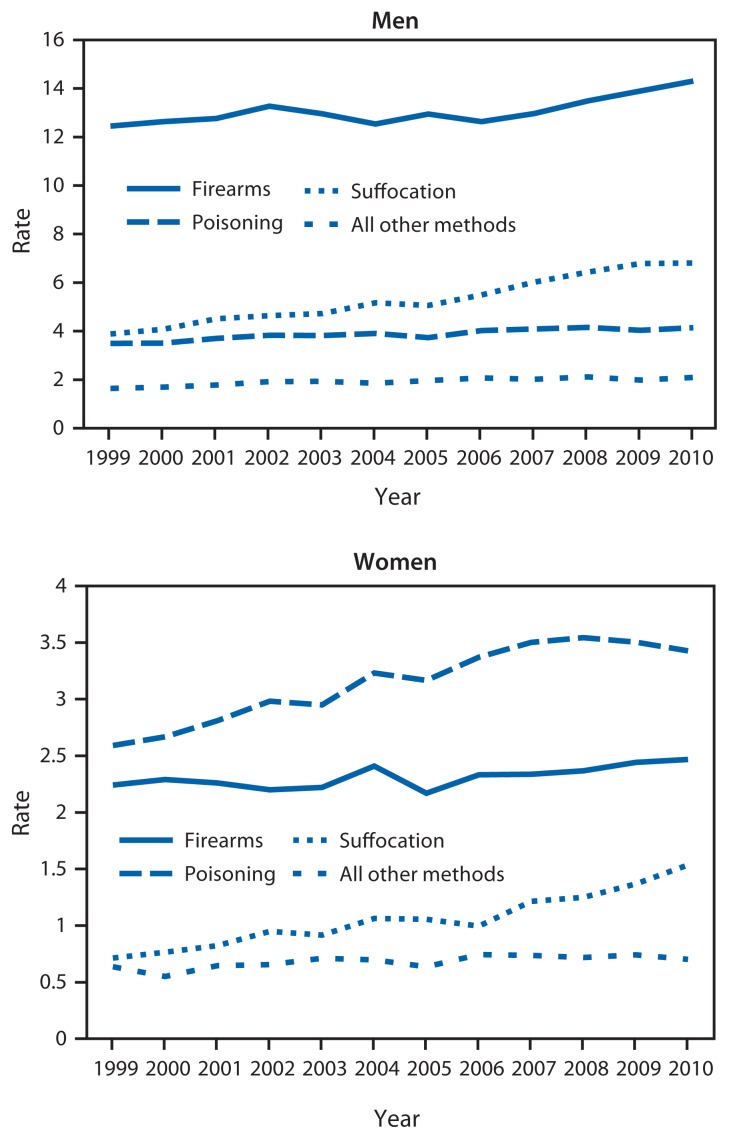
Trends in age-adjusted suicide rates^*^ among persons aged 35–64 years, by sex and mechanism — National Vital Statistics System, United States, 1999–2010 ^*^ Per 100,000 population.

**TABLE 1:** Number of suicides, age-adjusted suicide rates,* and percentage change in rates from 1999 to 2010 among persons aged 35–64 years, by selected characteristics — National Vital Statistics System, United States, 1999–2010

Characteristic	1999		2010		% change in rate	(95% CI)
	
	No.	Rate	No.	Rate		
**Total**	**14,443**	**13.7**	**21,754**	**17.6**	**28.4**	**(25.7–31.2)**
**Age group (yrs)**						
35–39	3,286	14.4	3,084	15.3	6.4	(1.3–11.8)
40–44	3,180	14.3	3,487	16.7	16.5	(11.0–22.2)
45–49	2,817	14.3	4,372	19.3	34.3	(28.1–40.8)
50–54	2,264	13.4	4,427	19.9	48.4	(41.1–56.1)
55–59	1,678	12.8	3,760	19.1	49.1	(40.8–57.9)
60–64	1,218	11.4	2,624	15.6	37.0	(28.0–46.6)
**Race/Ethnicity** †						
White	12,536	15.9	18,848	22.3	40.4	(37.2–43.6)
Black	772	6.4	970	6.8	5.8	(−3.8–16.3)
Hispanic	691	7.1	1,180	7.4	3.5	(−5.9–13.9)
A/PI	285	7.1	509	7.8	10.6	(−4.4–27.9)
AI/AN	90	11.2	171	18.5	65.2	(27.7–113.6)
Other/Unknown	69	—	76	—	—	—
**U.S. Census region** §						
Northeast	2,178	10.5	3,190	13.9	32.7	(25.6–40.2)
Midwest	3,084	12.7	4,609	17.3	35.6	(29.5–42.0)
South	5,532	14.8	8,396	18.4	24.4	(20.3–28.8)
West	3,649	15.8	5,559	19.5	23.6	(18.5–28.9)
**Mechanism**						
Firearm	7,634	7.2	10,393	8.3	14.4	(11.0–17.8)
Poisoning	3,202	3.0	4,722	3.8	24.4	(18.9–30.2)
Suffocation	2,412	2.3	4,934	4.1	81.3	(72.7–90.4)
Other	1,195	1.1	1,705	1.4	22.5	(13.7–32.0)

**Abbreviations:** CI = confidence interval; A/PI = Asian/Pacific Islander; AI/AN = American Indian/Alaska Native.

* Per 100,000 population. Rates were age adjusted for all categories except age group.

† Race/ethnicity was coded into six mutually exclusive categories: white, black, AI/AN, A/PI, Hispanic, and other/unknown. All persons categorized in the first four groups were non-Hispanic. Persons categorized as Hispanic might be of any race.

§ *Northeast:* Connecticut, Maine, Massachusetts, New Hampshire, New Jersey, New York, Pennsylvania, Rhode Island, and Vermont; *Midwest:* Illinois, Indiana, Iowa, Kansas, Michigan, Minnesota, Missouri, Nebraska, North Dakota, Ohio, South Dakota, and Wisconsin; *South:* Alabama, Arkansas, Delaware, District of Columbia, Florida, Georgia, Kentucky, Louisiana, Maryland, Mississippi, North Carolina, Oklahoma, South Carolina, Tennessee, Texas, Virginia, and West Virginia; *West*: Alaska, Arizona, California, Colorado, Hawaii, Idaho, Montana, Nevada, New Mexico, Oregon, Utah, Washington, and Wyoming.

**TABLE 2:** Number of suicides, age-adjusted suicide rates,* and percentage change in rates from 1999 to 2010 among persons aged 35–64 years, by sex and selected characteristics — National Vital Statistics System, United States, 1999–2010

Characteristic	Men						Women					
	
	1999		2010		% change in rate	(95% CI)	1999		2010		% change in rate	(95% CI)
			
	No.	Rate	No.	Rate			No.	Rate	No.	Rate		
**Total**	**11,128**	**21.5**	**16,635**	**27.3**	**27.3**	**(24.3–30.5)**	**3,315**	**6.2**	**5,119**	**8.1**	**31.5**	**(25.8–37.4)**
**Age group (yrs)**												
35–39	2,590	22.7	2,372	23.6	4.0	(−1.6–10.0)	696	6.1	712	7.0	15.8	(4.3–28.5)
40–44	2,429	22.1	2,661	25.6	15.9	(9.7–22.5)	751	6.7	826	7.9	17.3	(6.3–29.5)
45–49	2,152	22.3	3,375	30.1	35.2	(28.1–42.8)	665	6.7	997	8.7	30.2	(18.0–43.6)
50–54	1,702	20.6	3,358	30.7	49.4	(40.9–58.4)	562	6.5	1,069	9.4	44.7	(30.6–60.2)
55–59	1,284	20.3	2,859	30.0	47.8	(38.3–57.8)	394	5.8	901	8.9	52.5	(35.5–71.7)
60–64	971	19.1	2,010	24.9	30.2	(20.6–40.5)	247	4.4	614	7.0	59.7	(37.7–85.1)
**Race/Ethnicity** †												
White	9,599	24.5	14,379	34.2	39.6	(36.0–43.4)	2,937	7.4	4,469	10.5	41.9	(35.4–48.8)
Black	631	11.3	766	11.4	1.0	(−9.2–12.3)	141	2.2	204	2.7	23.0	(−0.7–53.0)
Hispanic	570	11.8	959	12.1	1.9	(−8.4–13.3)	121	2.5	221	2.8	9.6	(−12.5–37.1)
A/PI	207	10.9	346	11.4	4.7	(−12.0–24.5)	78	3.6	163	4.7	28.9	(−1.5–69.4)
AI/AN	67	17.0	122	27.2	59.5	(18.1–115.2)	23	5.7	49	10.3	81.4	(10.0–198.6)
Other/Unknown	54	—	63	—	—	—	15	—	13	—	—	—
**U.S. Census region** §												
Northeast	1,693	16.8	2,502	22.4	33.4	(25.0–42.0)	485	4.6	688	5.9	29.1	(14.8–45.2)
Midwest	2,387	20.0	3,544	26.8	34.4	(28.0–42.0)	697	5.7	1,065	7.9	38.6	(26.0–53.0)
South	4,253	23.3	6,386	28.7	23.1	(18.0–28.0)	1,279	6.7	2,010	8.6	28.6	(20.0–38.0)
West	2,795	24.3	4,203	29.7	22.1	(16.0–28.0)	854	7.4	1,356	9.5	28.6	(18.0–40.0)
**Mechanism**												
Firearm	6,431	12.4	8,830	14.3	14.9	(11.4–18.6)	1,203	2.2	1,563	2.5	10.1	(2.0–18.7)
Poisoning	1,815	3.5	2,540	4.1	18.5	(9.9–27.9)	1,387	2.6	2,182	3.4	32.3	(23.6–41.6)
Suffocation	2,029	3.9	4,002	6.8	75.0	(66.0–84.5)	383	0.7	932	1.5	115.0	(90.7–142.3)
Other	853	1.6	1,263	2.1	27.3	(15.2–40.7)	342	0.6	442	0.7	10.3	(−3.7–26.2)

**Abbreviations:** CI = confidence interval; A/PI = Asian/Pacific Islander; AI/AN = American Indian/Alaska Native.

* Per 100,000 population. Rates were age adjusted for all categories except age group.

† Race/ethnicity was coded into six mutually exclusive categories: white, black, AI/AN, A/PI, Hispanic, and other/unknown. All persons categorized in the first four groups were non-Hispanic. Persons categorized as Hispanic might be of any race.

§ *Northeast:* Connecticut, Maine, Massachusetts, New Hampshire, New Jersey, New York, Pennsylvania, Rhode Island, and Vermont; *Midwest:* Illinois, Indiana, Iowa, Kansas, Michigan, Minnesota, Missouri, Nebraska, North Dakota, Ohio, South Dakota, and Wisconsin; *South:* Alabama, Arkansas, Delaware, District of Columbia, Florida, Georgia, Kentucky, Louisiana, Maryland, Mississippi, North Carolina, Oklahoma, South Carolina, Tennessee, Texas, Virginia, and West Virginia; *West*: Alaska, Arizona, California, Colorado, Hawaii, Idaho, Montana, Nevada, New Mexico, Oregon, Utah, Washington, and Wyoming.

## Footnotes

* Race/ethnicity was coded into six mutually exclusive categories: white, black, AI/AN, Asian/Pacific Islander, Hispanic, and other/unknown. All persons categorized in the first four groups were non-Hispanic. Persons categorized as Hispanic might be of any race.

† The age-adjusted annual suicide rates per 100,000 population and the statistically significant increases from 1999 to 2010 for the 39 states were as follows: Alabama (19.4, 17.8%), Arizona (24.3, 15.8%), Arkansas (22.8, 54.2%), California (15.0, 17.0%), Colorado (24.9, 41.3%), Connecticut (14.5, 30.5%), Florida (21.4, 24.0%), Georgia (16.3, 24.5%), Hawaii (21.9, 61.2%), Idaho (27.1, 53.9%), Illinois (13.2, 18.7%), Indiana (19.5, 53.8%), Iowa (17.8, 41.7%), Kansas (19.9, 38.9%), Kentucky (21.7, 39.4%), Massachusetts (12.8, 32.3%), Michigan (18.2, 41.6%), Minnesota (16.0, 34.5%), Mississippi (20.1, 31.4%), Missouri (19.9, 26.2%), New Hampshire (17.8, 45.5%), New Jersey (11.8, 31.3%), New York (11.7, 41.7%), North Carolina (18.0, 22.1%), North Dakota (20.2, 70.5%), Ohio (16.9, 41.5%), Oklahoma (24.4, 34.4%), Oregon (27.3, 49.3%), Pennsylvania (17.4, 23.8%), Rhode Island (22.6, 69.1%), South Carolina (19.8, 36.6%), South Dakota (23.5, 48.0%), Tennessee (20.6, 21.8%), Texas (16.5, 23.7%), Utah (24.7, 27.6%), Vermont (19.8, 57.9%), West Virginia (21.0, 30.2%), Wisconsin (19.2, 33.9%), and Wyoming (31.1, 78.8%).

§ Additional information available at http://www.cdc.gov/violenceprevention/nvdrs.

¶ Additional information available at http://wonder.cdc.gov/wonder/help/cmf/sr02_148.pdf.

## References

[1] Rockett IR, Regier MD, Kapusta ND (2012). Leading causes of unintentional and intentional injury mortality: United States, 2000–2009. Am J Public Health.

[2] Baker SP, Hu G, Wilcox HC, Baker TD (2013). Increase in suicide by hanging/suffocation in the U.S., 2000–2010. Am J Prev Med.

[3] CDC Web-based Injury Statistics Query and Reporting System (WISQARS).

[4] Hu G, Wilcox HC, Wissow L, Baker SP (2008). Mid-life suicide: an increasing problem in U.S. whites, 1999–2005. Am J Prev Med.

[5] National Cancer Institute (2012). Joinpoint regression program.

[6] Reeves A, Stuckler D, McKee M, Gunnell D, Chang S, Basu S (2012). Increase in state suicide rates in the USA during economic recession. Lancet.

[7] Luo F, Florence C, Quispe-Agnoli M, Ouyang L, Crosby AE (2011). Impact of business cycles on US suicide rates, 1928–2007. Am J Pub Health.

[8] Phillips JA, Robin AV, Nugent CN, Idler EL (2010). Understanding recent changes in suicide rates among the middle-aged: period or cohort effects?. Public Health Rep.

[9] Breiding MJ, Wiersema B (2006). Variability of undetermined manner of death classification in the US. Inj Prev.

[10] US Department of Health and Human Services, Office of the Surgeon General and the National Action Alliance for Suicide Prevention (2012). National strategy for suicide prevention: goals and objectives for action.

